# 

**Published:** 1893-12

**Authors:** 


					﻿Vol XIII.
DECEMBER, 1893.
NO. 12.
THE
HOMEOPATHIC pHY^lClAfl,
A Monthly Journal of Homoeopathic Materia Medica
and Clinical Medicine.
“ He it a freeman whom truth makes free, and all are slaves beside.”
,l8eek the Truth: come whence it may, cost what it will.”
EDITED AND PUBLISHED BY
WALTER M. JAMES, M. D.
PHILADELPHIA :
No. 1125 8PRUCK STREET.
X» O-3ST D O K :
ALFRED HEATH A CO., Sole Agents fob Gbeat Britain,
114 Ebury Street, Eaton Square.
Yearly Subscription, $2.50, invariably in advance. Single numberSf 25 etc.
Enured at Um Philadelphia Peat-Offlee aa Seoood-CIaae Mai) Matter.
				

